# The effects of escitalopram on myocardial apoptosis and the expression of Bax and Bcl-2 during myocardial ischemia/reperfusion in a model of rats with depression

**DOI:** 10.1186/s12888-014-0349-x

**Published:** 2014-12-04

**Authors:** Yiming Wang, Hongming Zhang, Fangxian Chai, Xingde Liu, Michael Berk

**Affiliations:** Department of Psychiatry, Hospital Affiliated to Guiyang Medical University, Guiyang, Guizhou 550004 China; Department of Cardiology, The General Hospital of Jinan Military Region, Jinan, 250031 China; Department of Cardiology, Hospital Affiliated to Medical University, 28 Guiyi Street, Guiyang City, Guizhou 550004 China; IMPACT Strategic Research Centre, School of Medicine, Deakin University, Geelong, 3220 Australia; School of Public Health and Preventive Medicine, Monash University, Melbourne, 3004 Australia; Department of Psychiatry, The University of Melbourne, Parkville, VIC 3050 Australia; Orygen Youth Health Research Centre, The University of Melbourne, Parkville, VIC 3050 Australia; Florey Institute of Neuroscience and Mental Health, University of Melbourne, Level 3, Alan Gilbert Building, Parkville, VIC 3010 Australia

**Keywords:** Escitalopram, Depression, Myocardial infarction, Bax, Bcl-2

## Abstract

**Background:**

Major depressive disorder (MDD) is an independent risk factor for coronary heart disease (CHD), and influences the occurrence and prognosis of cardiovascular events. Although there is evidence that antidepressants may be cardioprotective after acute myocardial infarction (AMI) comorbid with MDD, the operative pathophysiological mechanisms remain unclear. Our aim was therefore to explore the molecular mechanisms of escitalopram on myocardial apoptosis and the expression of Bax and Bcl-2 in a rat model of depression during myocardial ischemia/reperfusion (I/R).

**Methods:**

Rats were divided randomly into 3 groups (n = 8): D group (depression), DI/R group (depression with myocardial I/R) and escitalopram + DI/R group. The rats in all three groups underwent the same chronic mild stress and separation for 21 days, at the same time, in the escitalopram + DI/R group, rats were administered escitalopram by gavage (10 mg/kg/day). Ligation of the rat’s left anterior descending branch was done in the myocardial I/R model. Following which behavioral tests were done. The size of the myocardial infarction was detected using 1.5% TTC dye. The Tunel method was used to detect apoptotic myocardial cells, and both the Rt-PCR method and immunohistochemical techniques were used to detect the expression of Bcl–2 and Bax.

**Results:**

Compared with the D and DI/R groups, rats in Escitalopram + DI/R group showed significantly increased movements and sucrose consumption (P < .01). Compared with the DI/R group, the myocardial infarct size in the escitalopram + DI/R group was significantly decreased (P < .01). Compared with the D group, there were significantly increased apoptotic myocardial cells in the DI/R and escitalopram + DI/R groups (P < .01); however compared with the DI/R group, apoptotic myocardial cell numbers in the escitalopram + DI/R group were significantly decreased (P < .01). Compared with the DI/R group, there was a down-regulated Bax:Bcl-2 ratio in the escitalopram + DI/R group (P < .01).

**Conclusions:**

These results suggest that in patients with AMI comorbid with MDD, there is an increase in pro-apoptotic pathways that is reversed by escitalopram. This suggests that clinically escitalopram may have a direct cardioprotective after acute myocardial infarction.

## Background

Cardiovascular disease is a major driver of mortality globally, and depression a major driver of morbidity. Coronary heart disease (CHD) is the most common type of cardiovascular disease, and acute myocardial ischemia (AMI) is the classically emergent expression of CHD with an attendant mortality risk. With the development of an increasing understanding of the biology-psychology-social medicine nexus, there is deeper understanding of the risk factors of CHD. There is a relationship between social and psychological factors with CHD and cardiac events, such that high incident rates of depression and anxiety have been found in CHD patients [1], and depression has been proved to be an independent risk factor for CHD, influencing the occurrence and prognosis of cardiovascular events [2]. The mortality rate in patients with CHD and depression is higher than that in patients with CHD only [3]. Our original study [4] suggested that active pro-apoptotic pathways might be involved in the nexus between myocardial infarction and depression. It is unclear whether antidepressant therapy should be included in the management of AMI patients complicated by depression. It is of clinical and theoretical importance to have effective preventive and treatment strategies for depressive patients complicated by AMI. This has the potential to both improve the survival rates of AMI patients and reduce the consumption of limited medical resources.

Currently emergency percutaneous coronary intervention (PCI) has become a routine clinical treatment for AMI, but patients receiving PCI treatment risk post-operative damage such as myocardial ischemia and reperfusion [5]. Although there are some data on the use of antidepressants in myocardial infarction (MI) comorbid with major depressive disorder [6], the pathophysiological processes are unclear. Escitalopram is a widely used antidepressant, but its effectiveness in AMI patients complicated with depression awaits further studies. Our aim was to explore the molecular mechanisms of escitalopram on myocardial apoptosis during myocardial ischemia/reperfusion in a rat model of depression, specifically the expression of Bax and Bcl-2. It was hoped this might illuminate mechanisms and treatment for AMI patients complicated by depression.

## Methods

### Subjects

Male Sprague-Dawley rats (n = 24, weighing 250 g ± 20 g) were used for the experimental procedures). Twenty-four SD rats were divided randomly into 3 groups, with 8 rats in each group; D group (depression group), D_I/R_ group (depression with myocardial ischemia/reperfusion) and escitalopram + D_I/R_ group (escitalopram + depression with myocardial ischemia/reperfusion). The rats in all three groups underwent the same chronic mild unpredictable stress and separation for 21 days following which open field and sucrose preference behavioral tests were conducted. At the same time, for the escitalopram + D_I/R_ group, distilled water with escitalopram (10 mg/kg/day) was administered once a day by gavage for 21 days, while the rats with depression were receiving I/R surgery. Five rats died during the IR procedure because of arrhythmias, these rats were excluded in the experiments. Animals were managed in accordance with the American Psychological Association (APA) ethical standards regarding the treatment of rats and the National Institute of Health and Guide for the Care and Use of Laboratory Animals (NIH Publications No. 80-23) revised in 1996, and the study was approved by the ethics Committee on the Guidelines for Animal Experiment at Guiyang Medical University, No: SCXK 2008-0001, provided by Guiyang Medial University Experimental Animals Center. Efforts were made to minimize the number of animals used and their suffering.

### Chronic mild stress model (depression)

In this study, the chronic mild unpredictable stress and separation model was used as a validated animal model of depression [7]. All rats were given the following stressors [8,9], consisting each week of repeated periods of confinement to a small (38 × 20 × 16 cm) cage, restraint (1 h), water deprivation (24 h), food deprivation (24 h), isolation (24 h), flashing light (3 h), forced cold water swimming (10 minutes) and were group-housed in a soiled cage overnight. Stressors were used daily in a random and unpredictable order for 21 days. Open-field test [10] and sugar water consumption experiments [11] were carried out 1 day before and 22 days after chronic mild unpredictable stress and separation to observe the behavior and anhedonic-like state of all rats.

### Open field test and sucrose consumption test

The behavior and anhedonic-like state of all rats were detected by the open field [10] and sucrose consumption tests [11]. The behavioral response to a new environment has been used as an indicator of emotional state. This includes assessment of horizontal movements (the total number of crossing squares) and vertical movements (grooming and rearing) during a five-minute period. A special white square was used (80 × 80 × 40 cm), which has 25 sectors with black stripes on the ground. Animals were put separately in the same central sector, and their activity was recorded during a five-minute period by an installed video camera. Observers analyzed the results of videotapes. Sucrose consumption is a rat model of an anhedonialike state [12]. Every cage was offered a bottle of water and a bottle of 1% sucrose water. Total sucrose consumption was measured after 60 minutes.

### Induction of myocardial ischemia/reperfusion

In this study, we used the myocardial infarction model described by Wu et al., which is a validated animal model of myocardial infarction [13]. The rats were placed on the operation table face-up after being anesthetized by abdominal injection of 10% chloral hydrate (3 ml/kg), connected with a small animal ventilator and manual ventilation was conducted at 50-60 times per minute and tidal volume 2 ml/100 mg. A thoracic incision of about 2.0 cm was made, and the base of the heart was exposed by retractors in the central region of the rib cage. The myocardial infarction model was induced by ligating the left anterior descending coronary artery 2 mm from the tip of the left auricle by polypropylene suture for 40 minutes. Rats were monitored by ECG (electrocardiogram) before and after coronary artery ligation. Ischemia was confirmed by ST-T segment elevation seen in ECG recordings performed on the rats [14]. After the myocardial surface became blanched, the animal was returned to its home cage. All rats were administered an analgesic (2 mg/kg) butorphanol tartrate, s.c. every 8 h during the 24 h after surgery and an antibiotic (10,000 IU penicillin G, i.m). Five rats died during the IR procedure because of arrhythmias, these rats were excluded in the experiments.

### Myocardial infarct size

Twenty-four hours after completion of the behavioral tests [4], the rats were sacrificed by decapitation. The heart was removed, the aorta was cannulated and washed with saline, the left anterior descending coronary artery was ligated again at the same site, and the aorta was infused with 2 ml of 0.5% Evan’s blue to determine the extent of the non-colored ischemic risk area. The myocardium was then bisected into two parts from the apex to the base along the left anterior descending coronary artery, which was frozen at -80°C for five minutes, and then sliced into 2 mm transverse sections and stained using 2,3,5-triphenyl tetrazolium chloride (1.5%TTC) and myocardial infarction size (×2) was thus confirmed [15].

### Preparation of tissue sample

At the end of the experiment, the rats were sacrificed with abdominal anesthesia and the hearts were harvested, cleansed with DEPC water, an adequate proportion of the tissue were obtained and kept in Trozil liquid tank awaiting RT-PCR testing. The hearts were then put in 4% paraform solution and fixed at 4°C for 6 hours. The hearts were cleansed again with 0.01 M PBS, dehydrated with ascending series of ethanol, cleared in xylene, embedded in paraffin, sliced continuously at 5 μm, awaiting myocardial cell apoptosis and immunohistochemistry testing.

### Myocardial cell apoptosis assay

Tunel apoptosis assay was carried out on myocardial cells, strictly following the kit instructions. Brown particles in the cell karyon were indicative of positive cells. Observation of apoptosis with light microscopy × 400 was performed and 10 sites were chosen. The apoptosis percentage out of the total number of myocardial cells was used as the Apoptotic index (AI).

### Quantitative measure of Bcl-2 and Bax protein by immunohistochemical staining

For Bax and Bcl-2 protein determination [16], immuno-histochemical staining kits for Bax, Bcl-2 were provided by Wuhan Boster Bioengineering Co., Ltd. (Wuhan, China). The sections (thickness of 4 μm) were de-waxed and rehydrated with freshly distilled water. At room temperature, samples underwent inactivation of endogenous peroxidase for 10 minutes and were then washed with distilled water for 2 minutes (three times), then immersed in 0.1 mol/L citrate buffer solution (pH = 6.0) and heated in a microwave oven until boiling (twice, with a 5-minute interval). After refrigerated flushing three times with phosphate buffered saline (PBS) for 2 minutes, antigen-retrieval buffers were added for 10 minutes and tissues were then flushed three times with PBS for 2 minutes. At room temperature, samples were non-specifically blocked with normal goat serum for 20 minutes. After removal of the goat serum, rabbit anti-Bax, Bcl-2 antibody was added (incubated for 60 minutes at 37°C). Biotinylated goat anti-rabbit antibody immunoglobulin G was applied for 20 minutes and flushed with PBS for 2 minutes (three times). The tissues were placed in a streptavidin-biotin-enzyme complex reagent and incubated for 20 minutes at 37°C. After flushing with PBS for 5 minutes (four times), samples were stained with diaminobenzidine for 25 minutes and washed for 3 minutes. Then samples were stained with hematoxylin, and were dehydrated and mounted for microscopic examination. Images of the sections were obtained (400 X) using the Image-Pro plus 4.5 software (Media Cybernetics, Silver. Spring, USA) with brown cytoplasmic staining under light microscopy indicating a positive reaction of Bax, Bcl-2. The myocardial infarction border zone was chosen using a 10 high power fields (400×), in which the mean number of bax and bcl-2 positive cells were obtained, the Bax:Bcl-2 ratio was also confirmed. Image Pro Plus was used for image analysis and IOD/area was used for semi-quantity analysis.

### Bcl-2 and Bax mRNA determination

① The extraction of total RNA in rat myocardium was performed strictly following the instruction of TRNzol RNA Reagent Kit (TIANGEN, Beijing, China). ② RNA reverse transcription into cDNA was performed following the instructions of the Invitrogen M-MLV cDNA kit. ③ 2 × Taq PCR Green Mix kit were used for PCR reaction assay. 5 μL DNA MarkerIwas used as the control and 1.5% agarose gel electrophoresis was performed on 5 μL Rt-PCR product.

### Statistical analysis

SPSS19.0 software was used for statistical analysis. The results were described using (mean ± SEM). One-way ANOVA was carried out on the basis of homogeneity of variance, multiple comparison between the groups was performed using S-N-K method, and *P* < .05 was regarded as statistically significant.

## Results

### Behavioral analysis

Compared with the D and D_I/R_ groups, rats in Escitalopram + D_I/R_ group showed significantly increased scores of both horizontal (P < .01) and vertical movement (P < .05). Total sucrose consumption was also significantly increased (P < .01) (Table 1).

**Table 1:** **The comparison of the Open- Field test scores and sucrose consumption of rats in different after experiment (mean ± SEM)**

**Group**	**Horizontal movement (scores)**	**Vertical movement (scores)**	**Sucrose consumption (g)**
D	53.18 ± 4.20	22.83 ± 2.34	32.25 ± 3.01
D_I/R_	51.57 ± 5.96	21.06 ± 2.62	30.34 ± 4.15
Escitalopram + D_I/R_	63.69 ± 6.11^▲^	27.51 ± 3.09*	38.73 ± 3.22^▲^

Note: compared with D, DI/R groups, ^▲^P < 0.01, *P < 0.05.

D group: depression group, D_I/R_ group: depression with myocardium ischemia/reperfusion, escitalopram + D_I/R_ group: escitalopram + depression with myocardium ischemia/reperfusion.

### Infarct size

The myocardial infarct risk area of the left ventricular area was 60 ± 2.5% (mean ± SEM) indicated by Evan Blue staining. The size of the myocardial infarct in the myocardial infarct risk area was different in three groups: (D group 0 ± 0%, D_I/R_ group 40.28 ± 6.71%; esctialopram + D_I/R_ group 26.35 ± 3.07%). Compared with the D group, myocardial infarct size in the D_I/R_ and esctialopram + D_I/R_ groups were significantly increased (*P* < .01). Compared with the D_I/R_ group, myocardial infarct size in the escitalopram + D_I/R_ group was significantly decreased (*P* < .01).

### The effect of escitalopram on myocardial cell apoptosis in depression during myocardial ischemia/reperfusion

Compared with the D group, the index of myocardial cell apoptosis in rats in the D_I/R_ and esctialopram + D_I/R_ groups were significantly increased (*P* < .01); Compared with the D_I/R_ group, the index of myocardial cell apoptosis in the escitalopram + D_I/R_ group was significantly decreased(*P* < .01) (Table 2).

**Table 2:** **The effect of escitalopram on myocardial cells apoptosis in rats with depression during myocardium ischemia/reperfusion (mean ± SEM, %)**

**Group**	**n**	**Myocardial cell apoptosis index**
D	8	3.08 ± 0.32
D_I/R_	8	37.14 ± 4.10*
Escitalopram + D_I/R_	8	28.87 ± 3.91*^▲^

Note: *P < .01 *vs* D group; ^▲^ *P* < .01*vs* D_I/R_ group.

D group: depression group, D_I/R_ group: depression with myocardium ischemia/reperfusion, escitalopram + D_I/R_ group: escitalopram + depression with myocardium ischemia/reperfusion.

### The effect of escitalopram on Bax and Bcl-2 mRNA and protein expression and ratio of Bax/Bcl-2 in myocardial cell in depression during myocardial ischemia/reperfusion

**Figure 1:**
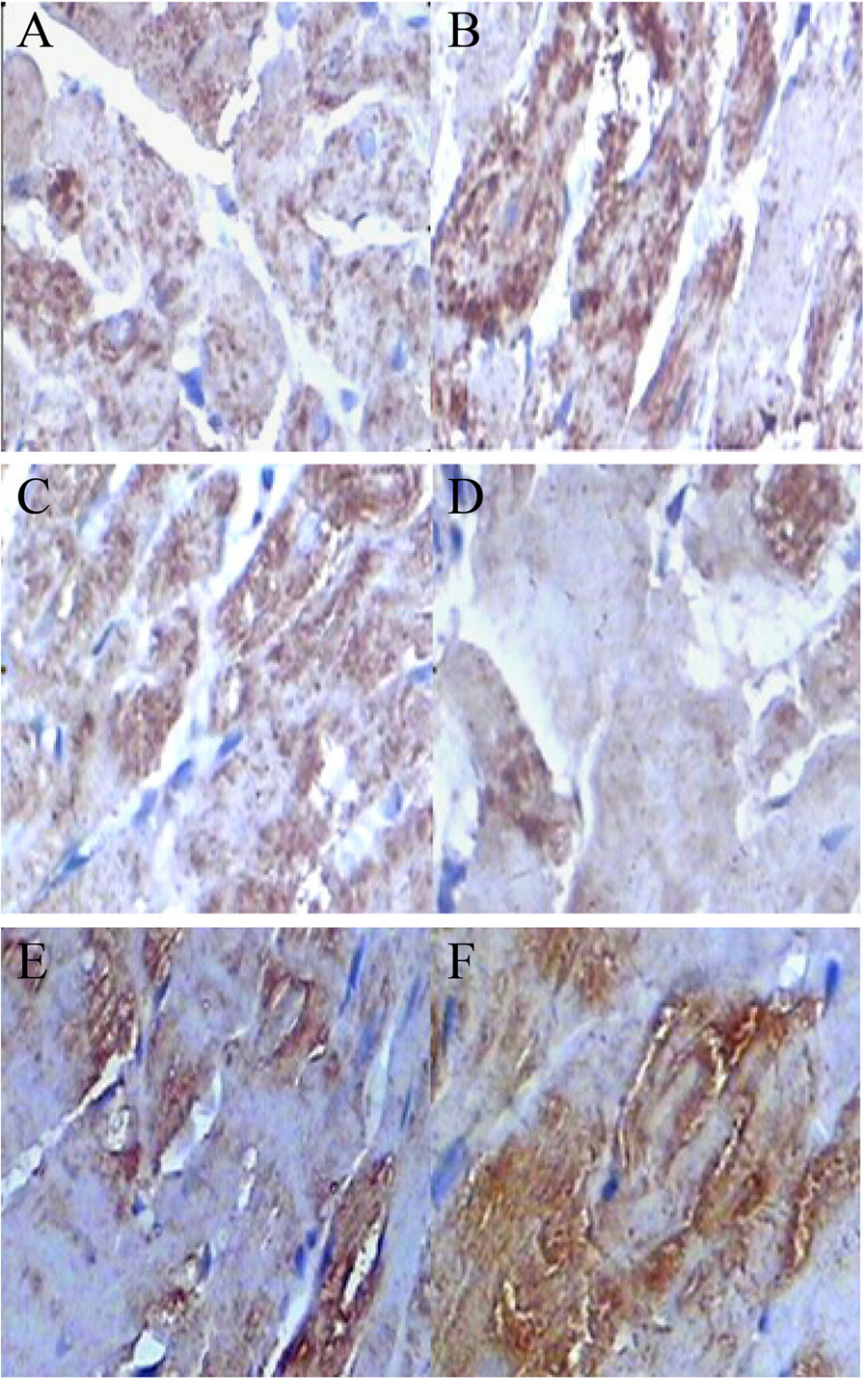
**The expression of protein Bax and Bcl-2 in myocardial cells (×400). A** Bax in depression group, **B** Bax in myocardium ischemia/reperfusion, **C** in escitalopram+depression with myocardium ischemia/reperfusion group, **D** Bcl-2 in depression group, **E** Bcl-2 in myocardium ischemia/reperfusion group, **F**: Bcl-2 in escitalopram + depression with myocardium ischemia/reperfusion group.

Compared with the D group, in the D_I/R_ group both Bax and Bcl-2 mRNA and protein expression were significantly increased (P < .01), however the ratio of Bax/Bcl-2 in the D_I/R_ group and the escitalopram + D_I/R_ group was significantly decreased (*P* < .01). Compared with the D_I/R_ group, in the escitalopram + D_I/R_ group Bax mRNA and protein expression was significantly decreased, while Bcl-2 mRNA and protein expression was significantly increased, and the ratio of Bax/Bcl-2 in escitalopram + D_I/R_ group was significantly decreased (*P* < .01) (Tables 3 and 4, Figure 1).

**Table 3:** **The effect of escitalopram on expression of mRNA Bax and Bcl-2 in myocardial cells in rats with depression during myocardium ischemia/reperfusion (mean ± SEM, copies/g RNA)**

**Group**	**Bax**	**Bcl-2**	**Bax/Bcl-2**
D	0.274 ± 0.0294	0.109 ± 0.0101	2.68 ± 0.14
D_I/R_	0.443 ± 0.0282*	0.398 ± 0.0216*	1.23 ± 0.25*
Escitalopram + D_I/R_	0.317 ± 0.0291^▲^	0.576 ± 0.0317^▲^	0.56 ± 0.28^▲^

Note: *P < .01 *vs* D group; ^▲^ *P* < .01*vs* D_I/R_ group.

D group: depression group, D_I/R_ group: depression with myocardium ischemia/reperfusion), escitalopram + D_I/R_ group: escitalopram + depression with myocardium ischemia/reperfusion.

**Table 4:** **The effect of escitalopram on expression of protein Bax and Bcl-2 in myocardial cells in rats with depression during myocardium ischemia/reperfusion (mean ± SEM, IOD)**

**Group**	**Bax**	**Bcl-2**	**Bax/ Bcl-2**
D	5.37 ± 0.42	2.17 ± 0.29	2.55 ± 0.21
D_I/R_	9.45 ± 0.76*	4.97 ± 0.74*	2.06 ± 0.22*
Escitalopram + D_I/R_	6.62 ± 0.38^▲^	6.18 ± 0.56^▲^	1.05 ± 0.18^▲^

Note: *P < .01 *vs* D group; ^▲^ *P* < .01*vs* D_I/R_ group.

D group: depression group, D_I/R_ group: depression with myocardium ischemia/reperfusion), escitalopram + D_I/R_ group: escitalopram + depression with myocardium ischemia/reperfusion.

## Discussion

### The effect of escitalopram on behaviour in depression during myocardial ischemia/reperfusion

In this study, we demonstrated that escitalopram improved the behavioral phenotype and anhedonic-like state in depression during myocardial ischemia/reperfusion, characterized by increased scores of horizontal movements, vertical movements and increased consumption of sucrose solution.

### The effect of myocardium ischemia/reperfusion on myocardial cell apoptosis, Bax and Bcl-2 expression in rats with depression

Since myocardial cells are non-renewable cells, myocardial cell apoptosis plays an important role in myocardial contractile dysfunction which may be present to a large extent when the heart is in a pathological situation, such as overload or myocardial ischemia [17]. Currently, percutaneous coronary intervention has become a main treatment for clinical coronary diseases, and plays a substantive role in revascularization strategies. But research has shown [18] that a sudden drop in blood pressure, cardiac insufficiency, arrhythmia, even sudden death can occur after percutaneous coronary intervention potentially, related to myocardial ischemia-reperfusion injury (MIRI). In MIRI, anoxic tissue injury occurs not just in acute ischemia, but after restoring blood perfusion. Myocardial cell apoptosis was considered one of the important factors in the pathogenesis of ischemia-reperfusion induced injury. The scale of apoptosis affects the gravity of MIRI.

The impact on the prognosis of coronary heart disease complicated by depressive disorder has attracted recent scholarly attention. Research has shown [19-21] that the incident rate of cardiovascular events increased significantly when coronary heart disease was complicated by comorbid depression. Wann [22] found depression in 15-30% of patients after myocardial infarction. Depression influences the onset, development and prognosis of coronary heart disease. Sertraline can prevent behavioural and biochemical markers in a rat model of following MI. Escitalopram can modify the behavioral syndrome through decreased pro-inflammatory cytokines and PGE2 in a rat model of post-cardiac infarct depression [23]; however the effects of antidepressants in this situation have not been well explored. This study showed that compared with depression group, the myocardial cell apoptosis index increased significantly in rats in the depression with myocardial ischemia/reperfusion group, suggesting that myocardial ischemia/reperfusion might cause increased myocardial cell apoptosis in rats with a depression phenotype. It is probable that in the case of patient suffering from AMI complicated by depression, the revascularization during percutaneous coronary intervention may result in myocardial cell apoptosis, potentially adversely impact cardiac function.

The mechanism whereby myocardial ischemia/reperfusion increases myocardial cell apoptosis in rats with depression remains unclear. Apoptosis is a type of genetically-regulated programmed cell death where apoptosis-related genes play key roles, probably including in chronic responsive stress-induced rat depression.

The Bcl-2 gene family [24-26] is considered the most important apoptosis-inhibiting genes, and include Bcl-2 and Bax. Their genetic products are mainly located on the mitochondrial membrane, karyotheca, and endoplasmic reticulum. Bcl-2 inhibits apoptosis while Bax stimulates it. This study showed that compared with the depression group, both Bax protein and mRNA expression increased significantly in myocardial cells in depression with myocardial ischemia/reperfusion, suggesting that myocardial ischemia/reperfusion stimulated myocardial cell apoptosis by upregulating Bax protein expression in myocardial cells of rats with depression. But this study also found that both Bcl-2 protein expression and mRNA expression were significantly higher in myocardial cells in rats with depression and myocardial ischemia than in the depression group, suggesting the apoptosis-inhibiting Bcl-2 gene was also involved in the process of ischemia/reperfusion inducing myocardial cell apoptosis in rats.

Bcl-2 is expressed through forming the Bcl-2 homology 1 and 2 (BH1 and BH2) domains by binding to Bax. When Bax expression is high, homodimer Bax/Bcl is formed to stimulate apoptosis, and when Bcl-2 expression is high, heterodimer Bcl-2/Bax is formed to inhibit the occurrence of apoptosis [27,28]. The regulatory effects of Bcl-2 and Bax on apoptosis is influenced not only by their expression respectively but also by the Bcl-2/Bax ratio. This study showed that in the depression with myocardial ischemia/reperfusion group, Bcl-2 expression increased significantly with an increase of Bax expression, his also might be caused by ischemia/reperfusion stimulating Bax expression and decreasing the formation of heter Bcl-2/Bax. This assumption still awaits further proof.

### Ischemia/reperfusion induced myocardial cell apoptosis and Bax, Bcl-2 expression in rats with depression

The role of antidepressant treatment in depression comorbid with CVD remains an unanswered question. In particular, the pathophysiological processes are unclear. 5-Hydroxytryptamine (5-HT) increases heart rate and atrial contraction through 5-HT4 receptors [29]. 5HT4 (a) and 5-HT4 (b) receptors are both expressed in the human atrium and ventricle. SSRIs influence heart function through decreasing heart rate and ameliorating left ventricular filling [30]. Escitalopram oxalate is the L-antimer of citalopram, a highly selective 5-HT reuptake inhibitor, whose inhibiting effect on 5-HT reuptake is twice that of racemic citalopram, and 100 times that of its d-isomer. It has low activity in inhibiting adrenalin and dopamine receptors. In the REMIT trial [31] escitalopram oxalate improved mental stress-induced myocardial ischemia defined as development of left ventricular ejection fraction reduction, regional wall motion abnormality and/or horizontal or down-sloping ST-segment depression, but the mechanisms remained unclear.

This study showed that the apoptosis index of myocardial cells decreased significantly in the escitalopram + ischemia/reperfusion group compared to the depression with myocardium ischemia/reperfusion group, suggesting that escitalopram might significantly decrease ischemia and reperfusion-induced apoptosis of myocardial cells in rats with a depression phenotype. This raises the question as to whether escitalopram oxalate may clinically improve heart function among patients with depression complicated with AMI, particularly after percutaneous coronary intervention. Furthermore, this study showed that compared with the ischemia/reperfusion group, Bax mRNA and protein expression decreased significantly, while Bcl-2 mRNA and protein expression increased significantly, moreover the Bax/Bcl-2 ratio decreased significantly, suggesting escitalopram may decrease ischemia/reperfusion induced apoptosis of myocardial cells in rats with a depression phenotype by upregulating Bcl-2 expression and down-regulating Bax expression.

This study has certain limitations that need to be kept in mind when interpreting these data. In this study, a control group of IR rats without pre-MI depression was lack. In our previous study (4), a control group of none-depressed animals with myocardial infarction (MI) was examined. We found that there were no behavioural signs of depression compared with depression group. Moreover in the post-MI depression group, there was a greater up-regulated Bax:Bcl-2 ratio compared with the MI group. The results of this animal model suggest that active pro-apoptotic pathways may be involved in the nexus between myocardial infarction and depression. In the current study, our aim was to explore the molecular mechanisms of escitalopram on myocardial apoptosis in a rat model of depression during myocardial ischemia/reperfusion (I/R), all rats were therefore deemed depressive rats, so we think that IR rats group without pre-MI depression is not necessary in this study, but this does require caution in the interpretation of the animal findings.

## Conclusions

These results suggest that in patients with myocardial infarction comorbid with major depressive disorder, there may be an increase in pro-apoptotic pathways that is potentially reversed by escitalopram. This suggests that escitalopram oxalate may have the potential to clinically improve heart function after patients with depression complicated with acute myocardial infarction after the percutaneous coronary intervention.

## Abbreviations

CHD: Coronary heart disease
AMI: Acute myocardial infarction
I/R: Ischemia/reperfusion
PCI: Percutaneous coronary intervention
APA: American Psychological Association
NIH: National Institute of Health
DEPC: Diethypyrocarbonate
RT-PCR: Reverse transcription polymerase chain reaction
PBS: Phosphate buffered saline
MIRI: Myocardial ischemia-reperfusion injury
BH1: Bcl-2 homology 1
BH2: Bcl-2 homology 1
